# Regulatory mechanisms of hepatocyte PCSK9 expression: translating mechanistic insights into potential nutraceuticals

**DOI:** 10.1186/s13020-025-01178-y

**Published:** 2025-08-05

**Authors:** Seon Kyeong Park, Jin-Taek Hwang, Hyo-Kyoung Choi, Jangho Lee

**Affiliations:** Precision Nutrition Research Group, Food Functionality Research Division, Korea Food Research Institute, Wanju-Gun, 55365 Republic of Korea

**Keywords:** PCSK9, LDL receptor, Nutraceuticals, Cholesterol metabolism, Cardiovascular disease

## Abstract

Proprotein convertase subtilisin/kexin type 9 (PCSK9) is a critical regulator of cholesterol metabolism in hepatocytes with profound implications for cardiovascular health. This review explores the intricate regulatory network that controls hepatic PCSK9 expression and explores how these molecular insights can be translated into nutraceutical applications. The precise control of PCSK9 involves complex interactions among transcription factors, signaling pathways, epigenetic modifications, and post-transcriptional mechanisms. Although pharmaceutical PCSK9 inhibitors demonstrated remarkable efficacy, their high cost has stimulated interest in natural alternatives. Bioactive compounds such as berberine, piceatannol, gallic acid, and organosulfur derivatives from garlic have demonstrated the ability to modulate PCSK9 expression through diverse mechanisms, often targeting the same molecular pathways as conventional drugs. These nutraceuticals not only inhibit PCSK9 but also enhance the expression and activity of the low-density lipoprotein receptor, offering a promising approach to reduce cardiovascular risk with potentially fewer side effects and greater accessibility. Understanding the precise mechanisms of these natural compounds advances the development of targeted dietary strategies to complement conventional pharmacotherapy in the treatment of hypercholesterolemia.

## Introduction

The discovery of proprotein convertase subtilisin/kexin type 9 (PCSK9) in 2003 marked a significant milestone in the understanding of cholesterol metabolism and cardiovascular disease. Initially identified by Seidah et al. as part of a gene family upregulated during apoptosis in neurons, PCSK9 was soon recognized for its pivotal role in lipid metabolism, particularly in the regulation of low-density lipoprotein receptors (LDLR) on hepatocytes [1, 2]. The identification of PCSK9 as the third gene responsible for autosomal dominant hypercholesterolemia was a groundbreaking discovery, as it linked gain-of-function mutations in PCSK9 to familial hypercholesterolemia, while loss-of-function mutations were associated with lower LDL cholesterol (LDL-C) levels and reduced cardiovascular risk [3, 4]. This understanding spurred the rapid development of PCSK9 inhibitors, a new class of lipid-lowering drugs that have shown remarkable efficacy in reducing LDL cholesterol levels by 50–60% and decreasing cardiovascular events [2, 3]. The clinical approval of monoclonal antibodies such as evolocumab and alirocumab in 2015, following successful trials, underscored the therapeutic potential of targeting PCSK9 [2, 3]. Beyond its role in cholesterol metabolism, PCSK9 has been implicated in various pathological processes, including atherosclerosis, inflammation, and even cancer, as it influences the degradation of several receptors beyond LDLR, such as CD36 and MHC-I, which are involved in fatty acid uptake and immune responses, respectively [5, 6].

PCSK9, a crucial regulator of cholesterol metabolism, is primarily synthesized in the liver, where it modulates plasma cholesterol levels by promoting the degradation of LDLRs, thus limiting hepatic clearance of circulating LDL-C [7, 8]. Beyond its canonical role in cholesterol metabolism, PCSK9 has been implicated in various aspects of hepatic pathophysiology, including lipid metabolism, inflammation, cellular migration, and cancer progression [7, 9]. PCSK9 inhibitors have demonstrated remarkable efficacy in reducing LDL-C levels and improving cardiovascular outcomes. Monoclonal antibodies (evolocumab and alirocumab) reduce LDL-C levels up to 60% and have shown significant reductions in cardiovascular events [10, 11]. Additionally, siRNA-based inclisiran achieves approximately 50% LDL-C reduction, though its cardiovascular outcome data are still being evaluated [12]. Emerging oral PCSK9 inhibitors, such as the macrocyclic peptide MK-0616 currently in phase III trials, represent another promising therapeutic approach [13]. While these pharmaceutical PCSK9 inhibitors have demonstrated substantial clinical efficacy, their high cost and limited accessibility highlight the potential value of natural compounds as complementary or alternative therapeutic strategies.

The precise control of PCSK9 expression in hepatocytes involves a complex interplay of transcriptional regulators, post-transcriptional mechanisms, and signaling pathways. Transcription factors such as HNF1α/β and SREBPs act as positive regulators of PCSK9 expression [14–16], whereas Farnesoid X Receptor (FXR) exerts inhibitory effects [17, 18]. In addition, microRNAs [19], histone modifications [20], and various signaling cascades, including the Janus kinase (JAK)/STAT [21], PI3K/Akt [22], and Suppressor of Cytokine Signaling-3 (SOCS-3) pathways [23], fine-tune PCSK9 levels in response to metabolic demands and environmental cues.

Although pharmaceutical PCSK9 inhibitors have demonstrated remarkable efficacy in reducing LDL-C levels, their high cost and limited accessibility have spurred interest in alternative approaches. Nutraceuticals—bioactive compounds derived from food sources—have emerged as promising alternatives for modulating PCSK9 expression with potentially fewer side effects and greater accessibility [24, 25]. Importantly, patients presenting with dyslipidemia typically exhibit multiple metabolic risk factors rather than isolated cholesterol abnormalities, including insulin resistance, hypertension, obesity, and hepatic steatosis [26]. Nutraceuticals offer distinct advantages in this context through their pleiotropic effects on metabolism, addressing multiple cardiovascular risk factors simultaneously [26]. For instance, bergamot polyphenolic fractions have demonstrated efficacy not only in reducing LDL-C and triglycerides but also in improving insulin sensitivity, reducing hepatic steatosis, and enhancing endothelial function [27]. Similarly, compounds containing chlorogenic acid and luteolin have shown beneficial effects on glucose metabolism, hepatic functionality, and vascular parameters in subjects with pre-obesity and metabolic syndrome [28]. This synergistic approach is particularly valuable given that nutraceutical combinations can result in enhanced bioavailability and action on multiple molecular targets, offering considerable advantages over single-compound interventions in managing the complex metabolic phenotype commonly observed in dyslipidemic patients [26]. Compounds such as berberine [25, 29], piceatannol [30], gallic acid [31], and organosulfur derivatives from garlic [32] can regulate PCSK9 through various mechanisms, often targeting the same molecular pathways as pharmacotherapy.

This review explores the intricate regulatory network that controls hepatocyte PCSK9 expression and examines how these molecular insights can be translated into nutraceutical applications. By elucidating the precise mechanisms through which natural compounds modulate PCSK9, more targeted and effective dietary strategies can be developed to complement conventional pharmacotherapy in the treatment of hypercholesterolemia and associated cardiovascular risks.

## Pathological roles of hepatic PCSK9

### Molecular mechanisms and therapeutic potential of PCSK9 in cholesterol regulation

PCSK9 plays a critical role in the regulation of cholesterol metabolism in hepatocytes by modulating the degradation of LDLR. PCSK9 binds to LDLR on the surface of hepatocytes, leading to its degradation in lysosomes, which impairs the clearance of circulating LDL-C from the plasma [5, 19, 33]. This mechanism is central to the pathophysiology of hypercholesterolemia and related cardiovascular diseases, as gain-of-function mutations in PCSK9 can lead to familial hypercholesterolemia, whereas loss-of-function mutations are associated with hypocholesterolemia [34, 35]. In the context of non-alcoholic fatty liver disease (NAFLD), PCSK9 is upregulated by treating with olanzapine, which enhances hepatic sterol regulatory element–binding protein 1c (SREBP-1c) expression, leading to increased cholesterol intake and lipid synthesis, thereby contributing to hepatic steatosis [36]. Furthermore, the interaction of PCSK9 with other proteins, such as CAP1 and HLA-C, influences its ability to degrade LDLR, with CAP1 enhancing the function of PCSK9 by stabilizing its closed conformation, which is necessary for targeting the PCSK9–LDLR complex to degradation compartments [37]. The therapeutic inhibition of PCSK9 through monoclonal antibodies or siRNA has proven effective in reducing LDL-C levels and the incidence of atherosclerosis, highlighting its potential as a target for treating hypercholesterolemia and possibly other liver-related pathologies [5, 33].

### Direct role of PCSK9 in LDLR degradation

PCSK9 plays a direct role in the degradation of LDLR in hepatocytes by binding to LDLR and promoting their lysosomal degradation, thereby preventing receptor recycling to the cell surface and reducing LDL-C uptake [38, 39]. This interaction is crucial for maintaining cholesterol homeostasis because PCSK9 binding to LDLR leads to the translocation of the complex to endosomes, where the acidic environment strengthens the binding affinity, preventing LDLR recycling [40]. Structural studies, including molecular dynamics simulations, have revealed that the variants of PCSK9 can significantly alter the binding affinity and structural flexibility of the LDLR–PCSK9 complex, which is essential for its degradation function [41, 42]. In addition, the C-terminal Cys/His-rich domain of PCSK9, particularly the M2 subdomain, is critical for LDLR degradation, potentially involving interactions with other proteins such as HLA-C, which may guide the complex to lysosomal compartments [37]. The role of PCSK9 in LDLR degradation is further supported by genetic studies showing that gain-of-function mutations in PCSK9 lead to reduced LDLR levels and increased plasma LDL-C, whereas loss-of-function mutations have protective effects against hypercholesterolemia [8]. These findings have driven the development of therapeutic strategies targeting PCSK9, such as monoclonal antibodies and siRNA therapies, which aim to inhibit its function and, enhance LDLR recycling and reduce LDL-C levels [38, 39]. Moreover, novel approaches like heterobifunctional molecules are being developed to accelerate PCSK9 clearance, offering alternative methods to mitigate its impact on LDLR degradation [43]. Interestingly, gender differences have been observed in the effects of PCSK9, with female mice exhibiting specific shedding of LDLR ectodomains, a process influenced by hepatic cholesterol levels and potentially modulated by estrogen [44]. Collectively, the direct role of PCSK9 in LDLR degradation is a pivotal mechanism in lipid metabolism, with important implications for cardiovascular health and therapeutic interventions.

### Mechanisms underlying statin resistance and the PCSK9 paradox

In the context of PCSK9 and hepatocytes, statin resistance involves complex interactions that affect cholesterol homeostasis and LDLR regulation [45]. Despite being effective in lowering LDL-C levels, statins paradoxically increase circulating PCSK9 levels, which can limit their efficacy by promoting LDLR degradation, thus reducing cholesterol clearance from the bloodstream [46]. The multichannel model of cholesterol regulation suggests that different lipoprotein particles are metabolized through distinct pathways within hepatocytes, which may explain the sustained increase in LDLR numbers despite the presence of statins and PCSK9 inhibitors [47]. In addition, statins induce PCSK9 expression through mechanisms involving geranylgeranyl isoprenoids and hepatic Rap1a, which regulate PCSK9 at the post-transcriptional level [46]. This induction can be mitigated by targeting Rap1a, which lowers PCSK9 levels and improves statin efficacy. Furthermore, statins suppress IDOL expression, another negative regulator of LDLRs, thereby contributing to increased LDLR expression and enhanced cholesterol clearance [48]. The combination of statins with PCSK9 inhibitors, such as the anti-PCSK9 antibody 1B20 has been shown to enhance the LDL-C-lowering effect, constituting a promising strategy for overcoming statin resistance and reducing cardiovascular risk [49]. This comprehensive approach highlights the potential of combined therapies to optimize cholesterol management and address the limitations of statin monotherapy.

### PCSK9 roles beyond cholesterol metabolism

PCSK9 plays multifaceted pathological roles in hepatocytes, affecting cholesterol metabolism, liver diseases, and cancer. Primarily synthesized in the liver, PCSK9 regulates cholesterol levels by promoting the degradation of LDLRs, thereby elevating circulating LDL-cholesterol [33, 50]. Beyond cholesterol homeostasis, PCSK9 is increasingly recognized as a contributor to chronic liver diseases. It influences inflammation, fatty acid metabolism, and carcinogenesis within hepatic tissues [33]. In hepatocytes, PCSK9 affects cellular processes such as migration and cholesterol efflux; for example, the gain-of-function mutation PCSK9-D374Y impairs these functions by modulating extracellular signal-regulated kinase activity [51]. In NAFLD, PCSK9 contributes to hepatic steatosis through receptor-dependent and -independent pathways, upregulating genes associated with lipid synthesis and cholesterol biosynthesis [36]. In hepatocellular carcinoma, PCSK9 exhibits dual roles: it can promote tumor growth by inhibiting apoptosis through the Bax/Bcl-2/Caspase pathway [52] and also acts as a tumor suppressor by interacting with GSTP1 and inhibiting the c-Jun n-terminal kinase signaling pathway [53]. In addition, PCSK9 influences metabolic dysfunction–associated steatotic liver disease, with its hepatic expression levels correlating with disease severity and affecting bariatric surgery outcomes [54]. In liver cancer cells, PCSK9 inhibition has been shown to induce metabolic exhaustion and cell death via ferroptosis, highlighting its potential as a therapeutic target [55]. Collectively, these studies underscore the complex and multifaceted roles of PCSK9 in hepatic pathophysiology, extending beyond its traditional role in cholesterol regulation to encompass broader implications in liver disease and cancer progression.

In addition to its hepatic functions, PCSK9 also plays important roles in vascular pathophysiology, the expression of PCSK9 in vascular wall cells is indeed directly related to the occurrence of atherosclerosis and aneurysms, as evidenced by multiple studies. In the context of atherosclerosis, PCSK9 is expressed by various cell types within the vascular wall, including endothelial cells, smooth muscle cells, and macrophages, and is found within atherosclerotic plaques [56]. This local expression of PCSK9 contributes to vascular inflammation and the progression of atherosclerosis through mechanisms that are both dependent and independent of LDLR pathways [57, 58]. Specifically, PCSK9 has been shown to induce senescence and apoptosis in vascular smooth muscle cells, which are critical processes in the development of degenerative vascular diseases such as atherosclerosis and aneurysms [59]. Furthermore, PCSK9's interaction with other receptors, such as CD36 and LRP-1, suggests its involvement in LDLR-independent pathways that exacerbate vascular inflammation and lesion formation [9]. Experimental models have demonstrated that PCSK9 can increase the infiltration of inflammatory monocytes into atherosclerotic lesions, further promoting inflammation and plaque instability [57]. These findings underscore the potential of PCSK9 as a therapeutic target not only for lowering systemic LDL levels but also for mitigating local vascular inflammation and the progression of vascular diseases [60]. Recent clinical studies in morbidly obese patients have revealed that PCSK9 plays a distinct role in liver fat accumulation independent of its effects on liver damage progression. Hepatic PCSK9 protein expression decreases significantly with increasing steatosis severity, while circulating PCSK9 levels positively correlate with the degree of hepatic fat accumulation [61]. This paradoxical relationship suggests that as liver steatosis progresses, increased PCSK9 secretion into the bloodstream occurs, which subsequently promotes LDL receptor degradation and elevates circulating cholesterol levels, thereby contributing to increased cardiovascular risk in obese populations. Importantly, PCSK9's hepatic expression correlates with genes involved in de novo lipogenesis, including FASN and PPARγ, indicating its direct involvement in lipid metabolism pathways rather than inflammatory processes [61]. Therefore, the expression of PCSK9 in vascular wall cells is intricately linked to the pathogenesis of atherosclerosis and aneurysms, highlighting its multifaceted role in cardiovascular biology.

## Regulators of PCSK9 expression in hepatocytes

**Fig. 1:**
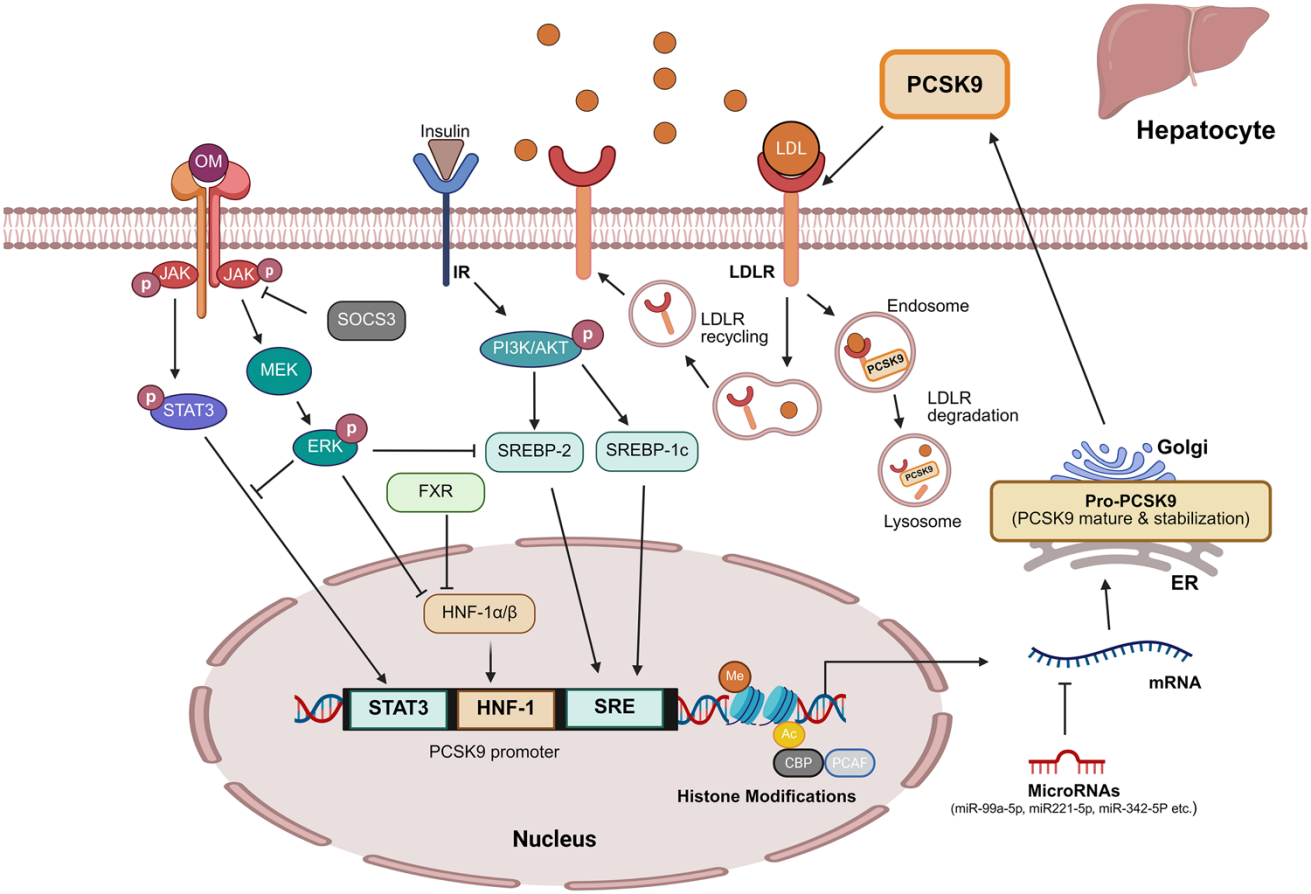
Regulatory Network of PCSK9 Expression in Hepatocytes. This illustration depicts the complex regulatory network of PCSK9 expression in hepatocytes, displaying a hepatocyte with a plasma membrane and nucleus along with the transcription of the PCSK9 gene, subsequent mRNA processing, and translation into the mature PCSK9 protein by various regulating factors. Transcription factors (HNF1α/β, SREBP-1c, and phosphorylated STAT3) increase PCSK9 expression by binding to specific elements in the PCSK9 promoter region. Conversely FXR, activated by bile acids, suppresses PCSK9 transcription. In signaling pathways, the activation of JAK1/JAK2 by Oncostatin M induces the MEK1/ERK pathway, which suppresses transcription factors and consequently downregulates PCSK9 expression. SOCS-3 promotes PCSK9 expression by inhibiting JAK/STAT3 phosphorylation. The activation of the PI3K/Akt pathway inhibits PCSK9 expression through SREBP-2 signaling modulation. Histone modifications such as H3K4me3 and H3K9 acetylation at the PCSK9 promoter region enhance the PCSK9 transcriptional activity. From the perspective of post-transcriptional regulation, microRNAs such as miR-99a-5p and miR-221-5p target the 3′-UTR of PCSK9 mRNA and suppress PCSK9 expression, thereby leading to increased LDLR recycling. Activation pathways are indicated by arrows (→), and inhibitory actions are represented by blocked lines (⊥), with this integrated network highlighting potential targets for therapeutic interventions aimed at modulating PCSK9 levels. Created with BioRender.com. Accessed on 09 April 2025

The expression and activity of PCSK9 in hepatocytes are tightly regulated by a complex network of transcriptional, post-transcriptional, and signaling regulators that respond to a variety of physiological and pathological stimuli [62]. A comprehensive understanding of these regulatory pathways is crucial for the development of targeted therapies for hypercholesterolemia and related cardiovascular diseases. This section examines the diverse regulators of PCSK9 expression in hepatocytes, including transcription factors, signaling pathways, epigenetic modifications, and post-transcriptional regulators. Table 1 and Fig. 1 provide a detailed summary of these regulator factors, their effects on PCSK9 expression, mechanisms of action, and physiological relevance, detailing their effects on PCSK9 levels and improving lipid metabolism through pharmacological and nutraceutical interventions.

**Table 1:** Regulators of PCSK9 Expression in Hepatocytes

Regulator	Effect on PCSK9	Mechanism of action	Significance	References
Transcription factors				
Hepatocyte nuclear factor 1 (HNF-1α/β)	Positive	• Binds to HNF1 binding site in PCSK9 promoter • Essential for statin-induced elevation of PCSK9	• Knockdown of HNF1α/β blunts statin-induced increase in PCSK9 levels	[14, 15, 48, 63, 96, 115]
Sterol regulatory element-binding protein-1c (SREBP-1c)	Positive	• Activates PCSK9 transcription through sterol regulatory elements (SREs) in promoter region • Regulated by insulin via PI3K pathway	• Modulated by dietary factors: - n-3 PUFAs reduce PCSK9 expression - Fructose increases PCSK9 expression	[16, 17, 49, 64, 65, 99]
Farnesoid X receptor (FXR)	Negative	• Natural (CDCA) and synthetic (GW4064) FXR agonists decrease PCSK9 expression	• Reduces LDLR degradation • Enhances clearance of LDL cholesterol	[17, 18, 62, 66, 67]
Signaling pathways				
Janus Kinase (JAK1/JAK2)	Negative	• Oncostatin M (OM) activates JAK • Suppresses PCSK9 via MEK1/ERK signaling pathway	• OM treatment reduces: - Liver PCSK9 mRNA by 69% - Protein by 41% in hypercholesterolemic hamsters	[21, 68, 69]
Suppressor of cytokine signaling-3 (SOCS-3)	Positive	• Inhibits STAT3 phosphorylation • Induces PCSK9 expression • Activates SREBP-1 pathway	• Retroviral overexpression: - Increases PCSK9 mRNA (3.48-fold) - Increases protein levels • Enhances de novo lipogenesis	[23, 70–72]
PI3K/Akt pathway	Negative	• H₂S and DADS inhibit PCSK9 via PI3K/Akt-SREBP-2 signaling	• Increases LDLR levels • Enhances lipid uptake without causing lipid accumulation	[22, 67, 73, 74]
Epigenetic regulators				
Histone modifications	Positive	• Statins increase: - H3K4me3 - H3K9 acetylation at PCSK9 promoter • Via SET1/COMPASS proteins and acetyltransferases	• Enhances transcriptional activity of PCSK9 • Potentially counteracts cholesterol-lowering effects of statins	[20, 63]
Post-transcriptional regulators				
MicroRNAs	Negative	• Multiple miRNAs target 3'-UTR of PCSK9 mRNA: - miR-99a-5p - miR-221-5p, miR-342-5p - miR-363-5p, miR-609 - miR-765, miR-3165 - miR-191, miR-222, miR-224	• Reduces PCSK9 expression • Increases LDLR expression • Enhances LDL-C uptake • Expression altered in patients undergoing PCSK9 inhibitor therapy	[19, 75–77, 79]
Long Non-coding RNAs				
LASER	Positive	• Binds to LSD1 in nucleus • Affects HNF-1α gene promoter methylation	• Positively correlated with plasma PCSK9 levels • Increased by statin treatment	[80]
lincRNA-p21	Negative	• Acts as molecular sponge for miR-221 • Induces PCSK9 promoter de-acetylation through SIRT1	• Inhibits atherosclerosis development • Regulates miR-221/SIRT1/PCSK9 axis	[81]

PCSK9: Proprotein convertase subtilisin/kexin type 9; LDLR: Low-density lipoprotein receptor; LDL-C: Low-density lipoprotein cholesterol; CDCA: Chenodeoxycholic acid; H₂S: Hydrogen sulfide; DADS: Diallyl disulfide; PUFAs: Polyunsaturated fatty acids; LASER: Long non coding RNA in Lipid Associated Single nucleotide polymorphism gEne Region; LSD1: Lysine-specific demethylase 1

### Hepatocyte nuclear factor 1 (HNF1)

HNF1 plays an important role in regulating the expression of PCSK9 in hepatocytes. HNF1, particularly its α and β isoforms, acts as a transcriptional regulator by binding to specific elements in gene promoters, including that of PCSK9, to modulate their expression [14, 15]. The transcription of PCSK9 is partially controlled by a HNF1 binding site located in the proximal region of its promoter, which is essential for the statin-induced increase in PCSK9 expression [15]. This regulation is important because PCSK9 is involved in LDLR degradation, thereby influencing LDL-C levels in the plasma [17, 18]. Studies have shown that the knockdown of HNF1α or HNF1β in hamsters can blunt the statin-induced increase in PCSK9 levels, suggesting that both isoforms are positive regulators of PCSK9 transcription [15]. In addition, HNF1α interacts with other transcription factors, such as SREBP2, which regulates PCSK9 expression through a sterol regulatory element in its promoter [16, 63]. This interaction highlights the complex regulatory network involving HNF1α in maintaining cholesterol homeostasis and highlights its potential as a target for the treatment of hypercholesterolemia and related cardiovascular diseases [36, 47].

### Sterol regulatory element–binding protein-1c (SREBP-1c)

SREBP-1c plays a relevant role in the transcriptional regulation of PCSK9 in hepatocytes by interacting with sterol regulatory elements (SREs) within the PCSK9 promoter region [16]. Along with SREBP-1c, SREBP-2 can activate the transcription of PCSK9 by binding to these SREs, as demonstrated in HepG2 cells where the presence of sterols influences PCSK9 expression through these transcription factors [16]. Insulin is a key regulator of SREBP-1c, enhancing its transcriptional activity in hepatocytes through the phosphatidylinositol 3-kinase pathway. This leads to increased levels of the nuclear mature form of SREBP-1c, thereby influencing the expression of lipid metabolism-related genes, including PCSK9 [64, 65]. The regulation of PCSK9 by SREBP-1c is also modulated by dietary factors; for instance, n-3 polyunsaturated fatty acids can reduce PCSK9 expression, whereas dietary fructose can upregulate it, indicating that SREBP-1c-mediated pathways are sensitive to nutritional inputs [17]. Furthermore, the use of PCSK9 inhibitors such as monoclonal antibodies can decrease the expression of SREBP-regulated genes, including PCSK9, suggesting the occurrence of a feedback mechanism where reduced PCSK9 activity alters the SREBP-1c activity and its downstream targets [49].

### Farnesoid X receptor (FXR)

FXR, a nuclear receptor activated by bile acids, plays a significant role in regulating PCSK9 expression in hepatocytes, which is crucial for maintaining cholesterol homeostasis. PCSK9, a serine protease, promotes the degradation of low-density lipoprotein receptors (LDLR) in the liver, thereby influencing plasma cholesterol levels [17, 18]. FXR, has been implicated in the transcriptional regulation of several genes involved in lipid metabolism, including PCSK9. Chenodeoxycholic acid, a potent natural FXR agonist, specifically decreases PCSK9 mRNA and protein levels in human hepatocytes by approximately 59%. Similarly, GW4064, a synthetic FXR agonist, has been shown to elicit comparable reductions in PCSK9 expression [66]. This regulatory effect is part of a broader metabolic network that includes other nuclear transcription factors such as SREBPs [17]. FXR activation can decrease PCSK9 expression, which in turn reduces LDLR degradation, thereby increasing the clearance of LDL-C from the bloodstream [62]. This mechanism is particularly relevant for therapeutic strategies aimed at lowering LDL-C levels, as PCSK9 inhibitors is known to effectively reduce cardiovascular risk by increasing LDLR availability [18, 62]. Moreover, the modulation of PCSK9 by FXR and other factors is not only crucial for cholesterol metabolism but also affects other pathways, such as inflammation and stress response, indicating a broader impact on hepatic and systemic metabolic processes [67].

### Janus kinase (JAK)

In their studies with oncostatin M (OM), a member of the interleukin-6 cytokine family, Cao et al. [21] demonstrated that JAK, specifically the JAK1 and JAK2 isoforms, plays a pivotal regulatory role in the suppression of PCSK9 expression in hepatocytes. When HepG2 cells were treated with OM, PCSK9 mRNA, and protein levels steadily decreased to approximately 40% of the control levels within 24 h, whereas the LDLR protein expression increased, suggesting that JAK activation plays a critical role in regulating cholesterol metabolism [21]. This function was confirmed by means of both pharmacological inhibition studies, where JAK inhibitor I and a JAK2-specific inhibitor prevented OM-mediated PCSK9 suppression, and siRNA-mediated silencing of JAK1 and JAK2, which restored PCSK9 expression in OM-treated cells [21, 68]. Notably, although OM induced transient phosphorylation of STAT proteins, the suppressive effect on PCSK9 was mediated by the MEK1/ERK signaling pathway downstream of JAK, as confirmed both in vitro and in hypercholesterolemic hamsters where OM treatment reduced liver PCSK9 mRNA by 69% and protein by 41% [21, 69].

### Suppressor of cytokine signaling-3 (SOCS-3)

SOCS-3 represents a critical regulatory pathway in the expression of PCSK9 in hepatocytes, as demonstrated by Ruscica et al. in their research on the interplay between inflammatory cytokines and lipid metabolism [23]. SOCS3, a negative regulator of the JAK/STAT pathway, induces PCSK9 expression at mRNA and protein levels in HepG2 cells, suggesting an important link between inflammation and cholesterol metabolism. This relationship was established through the retroviral overexpression of SOCS3 in HepG2 cells (HepG2SOCS3), which resulted in a considerable induction of PCSK9 mRNA (3.48-fold) and increased amounts of cellular and secreted PCSK9 protein [23]. The underlying mechanism most likely involves the inhibition of STAT3 phosphorylation, since SOCS3 overexpression completely abrogates basal STAT3 activation [23, 70]. Interestingly, this finding was confirmed through pharmacological inhibition using the STAT3 inhibitor MD77 and the JAK inhibitor JAK1, which produced similar effects on PCSK9 expression [23]. The physiological relevance of this regulatory pathway was validated in ob/ob mice, which exhibited higher hepatic levels of SOCS3 and correspondingly increased PCSK9 expression [23, 71]. Furthermore, SOCS3-induced PCSK9 expression was accompanied by the activation of the SREBP-1 pathway and enhanced de novo lipogenesis, leading to an increased apoB secretion and intracellular triglyceride accumulation [23, 72].

### PI3K/Akt signaling pathway

As a key component of the PI3K/Akt signaling pathway, Akt plays an important role in regulating PCSK9 expression in hepatocytes, particularly through its interaction with SREBP-2. The PI3K/Akt pathway is crucial for the modulation of lipid metabolism, as demonstrated in studies involving HepG2 cells [73]. For instance, hydrogen sulfide inhibits PCSK9 expression via the PI3K/Akt-SREBP-2 signaling pathway, which in turn increases the LDLR levels and enhances lipid uptake without causing lipid accumulation [22]. Similarly, diallyl disulfide, a compound derived from garlic, modulates lipid metabolism by inhibiting PCSK9 expression and promoting LDL uptake through the activation of the PI3K/Akt-SREBP2 pathway [73]. These findings highlight the importance of the PI3K/Akt pathway in the regulation of PCSK9 for modulating cholesterol levels in the blood [34, 74]. The regulation of PCSK9 by Akt and its downstream effects on SREBP-2 suggest a complex interplay that affects not only cholesterol metabolism but also other metabolic pathways in hepatocytes [67].

### Histone modifications

Histone modifications play a critical role in the regulation of PCSK9 expression in hepatocytes, particularly in response to statin treatment and the action of specific regulatory proteins. Atorvastatin, a commonly used statin, increases the enrichment of H3K4me3 and acetylation of H3K9 at the PCSK9 promoter in HepG2 cells by upregulating the expression of SET1/COMPASS proteins, such as SET1b and MLL1, and acetyltransferases like CREB-binding protein and P300/CBP-associated factor, which facilitate histone modifications [20]. These modifications are crucial as they enhance PCSK9 transcription, which can counteract the cholesterol-lowering effects of statins by increasing PCSK9 expression and subsequently reducing LDLR levels on hepatocytes [20]. In addition, SREBPs, particularly SREBP-2, are key transcription factors regulating PCSK9 by binding to SRE in its promoter. This interaction is further enhanced by Histone Nuclear Factor P, which, along with its cofactor NPAT, recruits histone acetyltransferase cofactors such as TRRAP to mediate histone H4 acetylation, thereby promoting PCSK9 transcription [63]. The sterol-dependent regulation of PCSK9 is predominantly mediated by SREBP-2, which is crucial for the transcriptional activation of PCSK9 in response to cholesterol levels [16].

### Non-coding RNAs (ncRNAs)

Non-coding RNAs (ncRNAs), including microRNAs (miRNAs), long non-coding RNAs (lncRNAs), and circular RNAs (circRNAs), have emerged as critical regulators of PCSK9 expression and cholesterol metabolism. These molecules provide an additional layer of post-transcriptional regulation that fine-tunes PCSK9 levels in response to various physiological and pathological conditions.

#### MicroRNAs (miRNAs) regulating PCSK9

In the regulation of PCSK9 expression in hepatocytes, several miRNAs have been identified as key modulators. MiR-99a-5p directly binds to the 3′-UTR of human PCSK9 mRNA, leading to a considerable downregulation of PCSK9 expression [19]. This interaction results in the upregulation of LDLR and enhanced uptake of LDL-C in human hepatocytes, suggesting its potential as a therapeutic target for hypercholesterolemia [19]. In addition, a high-throughput screening identified other miRNAs, including miR-221-5p, miR-342-5p, miR-363-5p, miR-609, miR-765, and miR-3165, which also target the 3′-UTR of PCSK9, reducing its expression and consequently increasing LDLR expression on hepatic cells [75]. Furthermore, miR-191, miR-222, and miR-224 interact with the 3′-UTR of PCSK9, decreasing the PCSK9 levels in HepG2 cells, which further supports their role in the post-transcriptional regulation of PCSK9 [76]. The expression of miR-191-5p and miR-224-5p was found to be altered in patients undergoing PCSK9 inhibitor therapy, indicating their involvement in the regulation of circulating PCSK9 levels [77]. Additional miRNAs have been identified that modulate PCSK9 expression through both direct and indirect mechanisms. MiR-27a reduces LDLR levels approximately 40% by direct binding to its 3′ non-coding region (3′UTR), and through indirect binding manner stimulates a threefold elevation in PCSK9 [78]. The PCSK9-C1420G haplotype has been linked to lower plasma PCSK9 and LDLC levels. Decourt et al. discovered in 2020 that the c.571C and c.234 T polymorphisms in the PCSK9 3′UTR are in close linkage disequilibrium with C1420G, and the variant containing c.571C had a 6.7 percent reduction in luciferase activity. Their findings showed that inhibiting hsa-miR-1228-3p and hsa-miR-143-5p had no impact on the haplotype displaying the c.*571C allele, indicating that hsa-miR-1228-3p and hsa-miR-143-5p reduce PCSK9 expression through targeting its 3′UTR [79].

#### Long non-coding RNAs (lncRNAs) and PCSK9 regulation

Long non-coding RNAs have emerged as important regulators of cholesterol homeostasis through their modulation of PCSK9 expression. LASER (long non coding RNA in Lipid Associated Single nucleotide polymorphism gEne Region) is highly expressed in both hepatocytes and peripheral mononuclear cells. Clinical studies showed that LASER expression is positively correlated with plasma PCSK9 levels in statin-free patients [80]. Mechanistically, LASER binds to LSD1 (lysine-specific demethylase 1) in the nucleus, and LASER knock-down enhances LSD1 targeting to genomic loci, resulting in decreased histone H3 lysine 4 mono-methylation at the HNF-1α gene promoter regions, subsequently affecting PCSK9 expression [80]. Another important lncRNA is lincRNA-p21, which acts as a molecular sponge for miR-221 to induce de-acetylation of the PCSK9 promoter sequence through SIRT1, thereby inhibiting atherosclerosis development [81].

#### Therapeutic implications of ncRNAs

The discovery of ncRNAs regulating PCSK9 has opened new therapeutic avenues for cholesterol management. Different ncRNAs play important roles in the progression of inflammatory atherosclerosis through targeting genes related to the PCSK9 pathway at the post-transcriptional level [82]. Statin treatment increased LASER expression, accompanied with increased PCSK9 expression, suggesting a feedback regulation that may partly explain statin escape during anti-cholesterol treatment. Therapeutic targeting of LASER might be an effective approach to augment the effect of statins on cholesterol levels in clinics [80]. Similarly, LincRNA-p21 shows promise as a therapeutic target for preventing atherosclerosis development through its regulation of the miR-221/SIRT1/PCSK9 axis [81].

## Therapeutic agents targeting PCSK9

PCSK9 inhibitors represent a revolutionary class of lipid-lowering therapeutics that have transformed cardiovascular disease management. Two fully human monoclonal antibodies, alirocumab and evolocumab, are currently approved for clinical use and demonstrate significant efficacy in reducing LDL-C and cardiovascular events [83]. PCSK9 inhibitors function by blocking the PCSK9 protein, which normally promotes degradation of LDL receptors on hepatocyte surfaces. Alirocumab and evolocumab modulate the upregulation of recycling and expression of LDL-C receptors at the cell surface, and increase LDL-C clearance from circulation [83]. By inhibiting PCSK9, these agents increase the number of LDL receptors available for cholesterol uptake, resulting in enhanced clearance of LDL-C from the circulation.

**Fig. 2:**
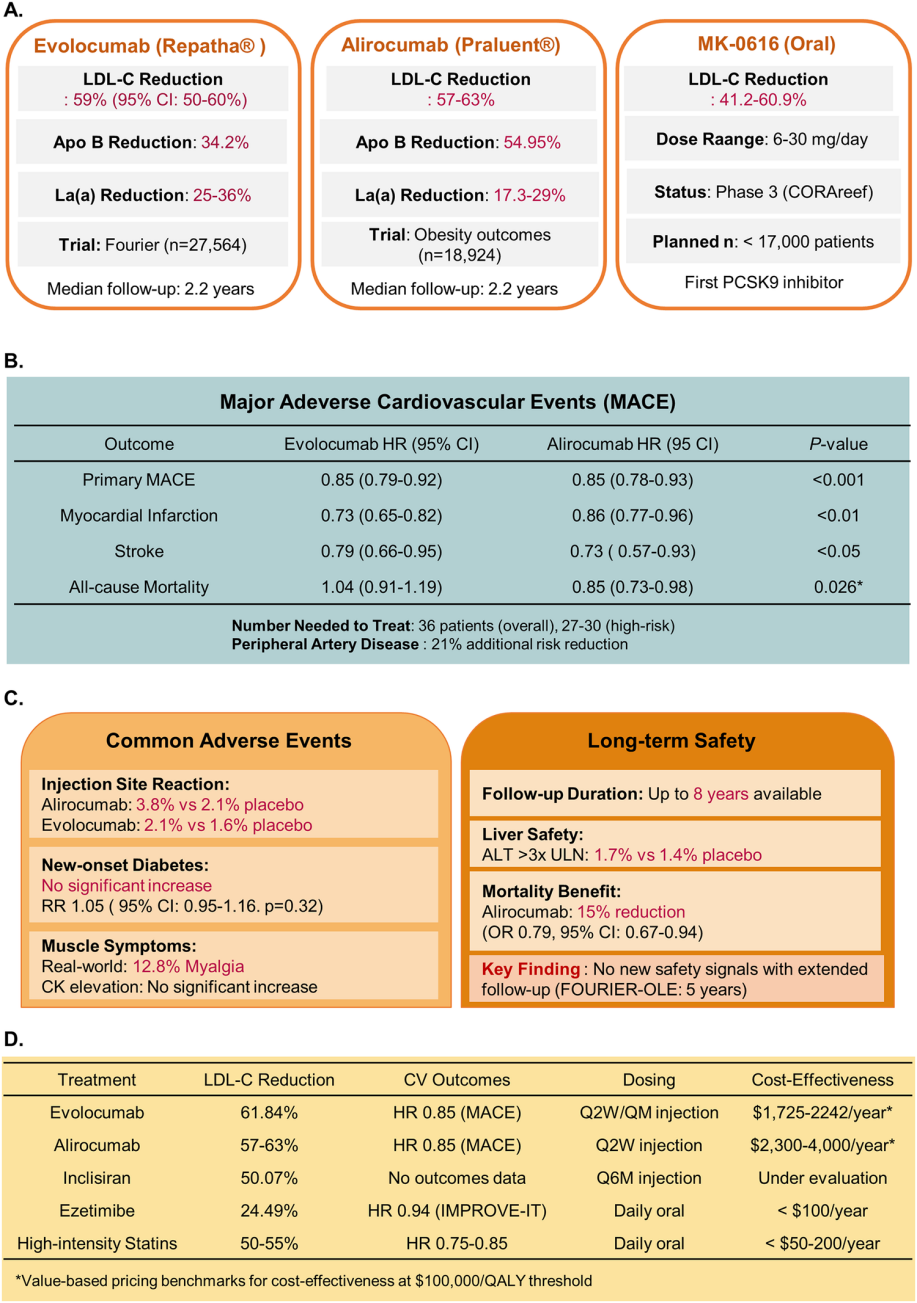
Clinical efficacy, safety profile, and comparative effectiveness of PCSK9 inhibitors in cardiovascular disease management. **A** Lipid-lowering efficacy of approved and investigational PCSK9 inhibitors. Data represent percentage reductions in low-density lipoprotein cholesterol (LDL-C), apolipoprotein B (Apo B), and lipoprotein(a) [Lp(a)] from baseline. Evolocumab data derived from the FOURIER trial (n = 27,564, median follow-up 2.2 years); alirocumab data from ODYSSEY OUTCOMES trial (n = 18,924, median follow-up 2.2 years); MK-0616 represents phase 3 data from the CORAref study. Values shown as percentages with 95% confidence intervals where available. **B** Cardiovascular outcomes from landmark randomized controlled trials. Hazard ratios (HR) with 95% confidence intervals (CI) for major adverse cardiovascular events (MACE), defined as the composite of cardiovascular death, myocardial infarction, stroke, unstable angina requiring hospitalization, or coronary revascularization. Data from FOURIER (evolocumab) and ODYSSEY OUTCOMES (alirocumab) trials. Number needed to treat (NNT) calculated for overall population and high-risk subgroups. *Asterisk indicates statistically significant mortality benefit observed exclusively with alirocumab treatment (P = 0.026). Peripheral artery disease risk reduction represents additional clinical benefit beyond primary MACE endpoint. **C** Safety and tolerability profile based on systematic review and meta-analysis of 39 randomized controlled trials encompassing > 66,000 patient-years of exposure. Left panel displays most frequent treatment-emergent adverse events with relative risk (RR) or odds ratio (OR) compared to placebo or standard care. Right panel presents long-term safety outcomes from extended follow-up studies (up to 8 years available data). Liver safety assessed by alanine aminotransferase (ALT) elevations > 3 × upper limit of normal (ULN). Mortality benefit data represent post-hoc analysis with 15% relative risk reduction. Creatine kinase (CK) elevations and myalgia rates derived from real-world pharmacovigilance data. **D** Head-to-head comparison of lipid-lowering therapies showing relative efficacy, cardiovascular outcomes, dosing frequency, and economic considerations. LDL-C reduction percentages represent pooled data from randomized trials. Cardiovascular outcomes expressed as hazard ratios for MACE where available. Dosing abbreviations: Q2W, every 2 weeks; QM, monthly; Q6M, every 6 months. Cost-effectiveness ratios calculated using incremental cost-effectiveness ratio methodology with quality-adjusted life years (QALY) as effectiveness measure. *Values represent healthcare economic evaluations based on $100,000 per QALY willingness-to-pay threshold from societal perspective. Apo B: apolipoprotein B; CI: confidence interval; CK: creatine kinase; HR: hazard ratio; LDL-C: low-density lipoprotein cholesterol; Lp(a): lipoprotein(a); MACE: major adverse cardiovascular events; NNT: number needed to treat; OR: odds ratio; PCSK9: proprotein convertase subtilisin/kexin type 9; QALY: quality-adjusted life years; RR: relative risk; ULN: upper limit of normal

Both alirocumab and evolocumab demonstrate substantial LDL-C lowering effects. Pooled results showed significant efficacy of evolocumab/alirocumab in reducing low-density lipoprotein cholesterol with a weighted mean difference of − 37.92% (95% confidence interval − 43.06% to − 32.78%) [84]. A recent comparative analysis showed that alirocumab 75 mg was superior to inclisiran 300 mg in reducing LDL-C in 24 weeks, achieving reductions of approximately 51.54% compared to 41.34% with inclisiran [85]. Beyond LDL-C reduction, PCSK9 inhibitors provide comprehensive lipid profile improvements **(**Fig. 2A**)**. These agents significantly reduced apolipoprotein B (weighted mean difference − 33.67%, 95% CI − 38.12% to − 29.22%) and lipoprotein(a) (weighted mean difference − 16.94%, 95% CI − 26.20% to − 7.69%) [84]. Meta-analysis data demonstrate that alirocumab/evolocumab showed significant efficacy in reducing Lp(a) with a weighted mean difference of – 20.10% (95% confidence interval – 25.59% to – 14.61%) **(**Fig. 2A**)** [86].

The cardiovascular benefits of PCSK9 inhibitors extend beyond lipid lowering. In randomized controlled trials, alirocumab and evolocumab have been reported to reduce the risk of recurrent cardiovascular disease in patients following an acute coronary event and secondary prevention populations when added to background statin therapy [83]. Importantly, alirocumab reduced risk of peripheral artery disease events (hazard ratio 0.69, 95% CI 0.54–0.89; P = 0.004), with benefits particularly pronounced in patients with elevated lipoprotein(a) levels **(**Fig. 2B**)** [87].

PCSK9 inhibitors demonstrate favorable safety profiles across diverse patient populations. Analysis suggests that alirocumab and evolocumab are generally safe and well tolerated and that their addition to background lipid-lowering therapy is not associated with an increased risk of adverse events or toxicity. However, some differences exist between agents. Alirocumab treatment significantly reduced the risk of serious adverse events compared to control treatment (risk ratio 0.937; 95% confidence interval 0.896–0.980), while no significant difference was observed with evolocumab treatment [83] **(**Fig. 2C**)**. Additionally, evolocumab showed early worsening of diabetes mellitus (OR 2.3, 95% CI 1.26 to 4.2, p = 0.041), particularly in the initial weeks of treatment, though this effect diminished with longer therapy duration [88]. The most common adverse effect is injection site reactions, with PCSK9 inhibitors associated with local injection site reactions (OR 1.54, 95% CI 1.37 to 1.73, p < 0.01), driven primarily by alirocumab use [88] **(**Fig. 2C**)**. In pediatric populations with familial hypercholesterolemia, PCSK9 inhibitors show promise, with evolocumab/alirocumab significantly reducing LDL-C and other lipid parameters while being generally well tolerated [84]. When compared to other lipid-lowering agents, PCSK9 inhibitors demonstrate superior efficacy **(**Fig. 2D**)**. Alirocumab was superior to inclisiran in improving the lipid profile, especially in reducing LDL-C, total cholesterol and triglycerides [85]. Beyond lipid lowering, PCSK9 inhibitors demonstrate beneficial effects on atherosclerotic plaque. The addition of PCSK9 inhibitors to statin therapy showed similar impact to ezetimibe in reducing plaque and lipid burdens, though more evidence is needed to confirm plaque composition effects **(**Fig. 2D**)** [89].

## Natural compounds that modulate PCSK9 in hepatocytes

**Fig. 3:**
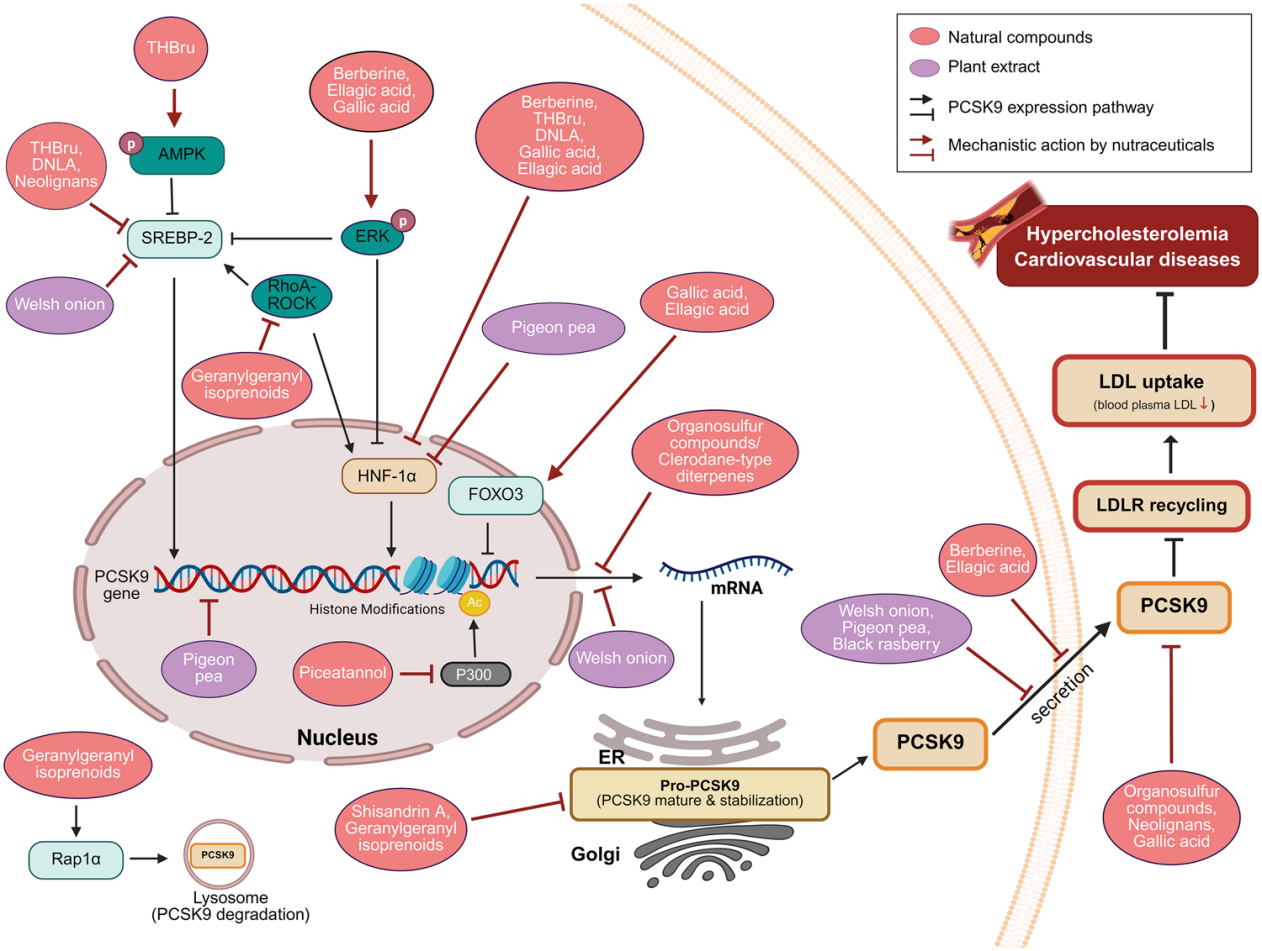
Mechanistic Actions of Key Nutraceuticals in PCSK9 Modulation. This figure summarizes the mechanisms by which various natural compounds and plant extracts modulate PCSK9 expression and activity in hepatocytes. Natural compounds (berberine, THBru, DNLA, gallic acid, ellagic acid, and Neolignans) and plant extracts (Welsh onion and pigeon pea) modulate the PCSK9 transcription factors such as HNF1α, SREBP2, and FoxO3. From the pathway perspective, natural compounds suppress PCSK9 expression by regulating the AMPK pathway (THBru), ERK1/2 pathway (berberine, ellagic acid, and gallic acid) and RhoA-ROCK pathway (geranylgeranyl isoprenoids). Piceatannol inhibits p300 histone acetyltransferase, thereby inhibiting PCSK9 expression. In addition, nutraceuticals (organosulfur compounds and clerodane-type diterpenes and Welsh onion extract) inhibit PCSK9 mRNA expression. Schisandrin A and geranylgeranyl isoprenoids inhibit the maturation and stabilization of PCSK9 protein. Nutraceuticals (berberine, ellagic acid, Welsh onion extract, pigeon pea extract, and black raspberry extract) inhibit the secretion of PCSK9 protein into the extracellular space, where it activates LDLR recycling. Geranylgeranyl isoprenoids also activate Rap1α, promoting PCSK9 degradation. These nutraceuticals enhance the reduction of PCSK9-mediated degradation of LDLR, thereby leading to hepatic LDL-cholesterol uptake. These regulatory actions may act as potential complementary or alternative approaches for managing hypercholesterolemia and related cardiovascular diseases. Activation pathways are indicated by arrows (→), and inhibitory actions are represented by blocked lines (⊥). Created with BioRender.com. Accessed on 09 April 2025

PCSK9 has emerged as a key therapeutic target in cholesterol management due to its role in regulating LDLR degradation and thereby increasing circulating LDL-C levels. Although pharmaceutical PCSK9 inhibitors, such as monoclonal antibodies, have demonstrated substantial clinical efficacy, there is growing interest in natural compounds that can modulate PCSK9 expression or activity with fewer side effects and greater accessibility [90, 91]. This section explores a variety of phytochemicals and plant extracts that exhibit PCSK9-inhibitory properties through various mechanisms, including transcriptional regulation, post-translational modification, and protein stability modulation. In addition to suppressing PCSK9, many of these natural compounds also upregulate LDLR expression and enhance its functional activity, promoting more efficient LDL-C clearance and reducing cardiovascular risk. Importantly, several of these compounds have been shown to counteract statin-induced upregulation of PCSK9, suggesting potential as adjunctive therapies that enhance the lipid-lowering effects of existing pharmacological treatments. Table 2 and Fig. 3 provide a comprehensive summary of these natural PCSK9 modulators, detailing their sources, molecular mechanisms, and biological effects, highlighting diverse approaches by which plant-derived compounds can influence cholesterol homeostasis through the PCSK9–LDLR axis. For this review, natural compounds were selected based on the following criteria: (1) documented evidence of PCSK9 modulation in peer-reviewed literature published between 2008 and 2025; (2) availability of mechanistic data demonstrating direct or indirect effects on PCSK9 expression, activity, or stability; (3) evidence from either in vitro hepatocyte studies or in vivo animal/human studies; and (4) compounds with established safety profiles and potential for clinical translation. We focused primarily on compounds with well-characterized molecular mechanisms and excluded those with limited or conflicting evidence. This selection approach ensures a comprehensive yet focused examination of the most promising natural PCSK9 modulators while acknowledging that the rapidly expanding field may contain additional candidates not covered in this review.

**Table 2:** Natural PCSK9 modulators: mechanisms and biological effects

Compound	Source	Mechanism of action	Biological effects	References
Organosulfur compounds	Garlic	• Inhibit PCSK9 activity • Downregulate PCSK9 mRNA expression	• Enhance LDL receptor function • Reduce circulating LDL cholesterol • Improve lipid profiles • Reduce inflammation	[32, 92–94]
Berberine	Goldenseal, Barberry, Oregon grape	• Reduce PCSK9 expression and secretion • Downregulate PCSK9 via ERK1/2 pathway • Modulate HNF1α and SREBP-2	• Enhance LDLR expression • Reduce TC, LDL, and TG • Increase HDL levels • Attenuate atherosclerotic lesions • Reduce hepatic steatosis	[25, 29, 95–99]
Tetrahydroberberrubine (THBru)	Berberine derivative	• Activate AMPK/SREBP2/PCSK9/LDL pathway • Directly bind to AMPK (binding energy: −6.6 kcal/mol) • Downregulate SREBP2 and PCSK9 expression	• Alleviate hyperlipidemia and obesity • Decrease serum TC, TG, and LDL-C • Upregulate LDL receptor levels • Enhance hepatic LDL clearance • Reduce statin-induced PCSK9 expression	[100–102]
Piceatannol (PT)	Grapes, berries, peanuts	• Inhibit p300 histone acetyltransferase (HAT) • Bind to p300 HAT domain (IC50: 19.89 μM) • Block p300 recruitment to PCSK9 promoter • Epigenetically regulate PCSK9 expression	• Suppress statin-induced PCSK9 expression • Stabilize LDLR expression • Improve cholesterol clearance • Reduce plasma LDL-cholesterol • Enhance hepatic LDLR stabilization	[30, 48, 103]
Gallic acid (GA)	Fruits, vegetables, tea	• Inhibit PCSK9 mRNA expression and protein • Activate FOXO3 and suppress HNF1α • Directly bind to PCSK9 active pocket (−5.36 kcal/mol) • Activate EGFR-ERK1/2 pathway	• Enhance LDLR protein accumulation • Extend LDLR mRNA half-life (0.59 h to 1.02 h) • Promote LDL uptake • Improve cholesterol metabolism • Manage NAFLD	[31, 104–108]
Ellagic acid	Fruits, vegetables, nuts	• Reduce PCSK9 expression and secretion • Inhibit HNF1α • Promote FoxO3 expression	• Upregulate LDLR protein levels • Decrease aortic plaque deposition • Reduce hepatic lipid accumulation • Ameliorate atherosclerosis	[105, 109]
Schisandrin A	Schisandra chinensis	• Inhibit PCSK9 protein stabilization • Destabilize free PCSK9 protein in proteasome • Protect LDLR from lysosomal degradation	• Increase LDLR expression • Enhance LDL uptake • Reduce serum lipid profiles • Attenuate increases in body weight • Reduce hepatic injury markers	[110, 111]
Geranylgeranyl isoprenoids	Mevalonate pathway derivatives	• Activate Rap1a • Inhibit RhoA-ROCK pathway • Enhance lysosomal degradation of PCSK9 • Post-transcriptionally regulate PCSK9 stability	• Suppress statin-induced PCSK9 elevation • Enhance LDL-C lowering • Potentially enhance statin efficacy	[46, 112]
Dendrobium nobile Lindl. alkaloids (DNLA)	Traditional Chinese herb	• Downregulate PCSK9 expression • Inhibit SREBP2 and HNF1α • Modulate LXRα/IDOL/LDLR pathway • Suppress LXRα expression	• Increase LDLR expression • Promote LDLR recycling • Decrease HMGCR • Increase CYP7A1 • Reduce statin-induced PCSK9 expression	[118]
Clerodane-type diterpenes	Casearia grewiifolia	• Inhibit PCSK9 mRNA expression	• Potential lipid-lowering effects	[119]
Lindenane sesquiterpenoid dimers	Chloranthus japonicus	• Reduce PCSK9 levels • Particularly effective: shizukaol F and chlorahololide D (69.0% and 72.8% inhibition at 5 μM)	• Increase LDL uptake • Upregulate LDLR expression • Demonstrate lipid-lowering effects • Show anti-inflammatory properties	[120]
Neolignans	Penthorum chinense	• Downregulate PCSK9 activity • Dibenzoxepine-type lignan (IC50: 5.13 μM) • Penthorin A (IC50: 15.56 μM) • Methyl gallate (IC50: 11.66 μM) • Upregulate LDLR via SREBP2	• Increase LDL-cholesterol uptake • Influence PPARγ-LXR-ABCA1 pathway • Affect cholesterol efflux • Regulate multiple genes in cholesterol metabolism	[121]
Welsh onion extract (WOE)	Welsh onion	• Attenuate PCSK9 and SREBP2 expression • Contain kaempferol and p-coumaric acid • Inhibit PCSK9 transcription and protein expression	• Prevent LDLR degradation • Stabilize LDLR protein • Maintain LDLR levels under lipid depletion	[110, 113]
Pigeon pea extract (MECC)	Cajanus cajan L. leaves	• Decrease PCSK9 mRNA and mature protein (up to 54%) • Inhibit PCSK9 promoter activity • Reduce nuclear HNF-1α levels • Contain cajaninstilbene acid as active component	• Increase LDLR mRNA and protein • Enhance cell-surface LDLR • Improve LDL uptake by up to 43% • Show selective gene regulation	[114, 115]
Black raspberry extract (BRE)	Black raspberry	• Suppress PCSK9 expression • Counteract statin-induced PCSK9 increase	• Enhance LDLR expression • Promote LDL uptake • Lower serum LDL cholesterol • Modulate gut microbiota and bile acid profiles • Improve cardiovascular health markers	[116, 117]

PCSK9: Proprotein convertase subtilisin/kexin type 9; LDLR: Low-density lipoprotein receptor; LDL-C: Low-density lipoprotein cholesterol; TC: Total cholesterol; TG: Triglycerides; HDL: High-density lipoprotein; SREBP: Sterol regulatory element-binding protein; HNF1α: Hepatocyte nuclear factor 1α; AMPK: AMP-activated protein kinase; FoxO3: Forkhead box O3; NAFLD: Non-alcoholic fatty liver disease

### Organosulfur compounds (OSCs)

Garlic-derived OSCs have shown potential in regulating PCSK9, thereby offering therapeutic benefits for cardiovascular health. Studies have demonstrated that compounds such as *S*-ethyl-l-cysteine, *S*-allyl-l-cysteine, and diallyl trisulfide effectively inhibit PCSK9 activity, as evidenced by their low in vitro IC50 values, suggesting their potential to enhance LDLR function and reduce circulating LDL-C levels [32]. Furthermore, these OSCs ameliorate metabolic syndrome-related conditions in animal models by downregulating PCSK9 mRNA expression and upregulating LDLR expression, thereby improving lipid profiles and reducing inflammation [92]. In addition, garlic-derived OSCs like propyl-propane thiosulfonate and propyl-propane thiosulfinate exhibit anti-inflammatory properties, which could further contribute to cardiovascular health by modulating immune responses and reducing oxidative stress [93]. The modulation of cholesterol biosynthesis by OSCs, particularly allicin, which inhibits the early steps of the pathway, further underscores their role in managing cholesterol levels [94].

### Berberine

Berberine, a natural isoquinoline alkaloid, has shown efficacy in controlling PCSK9 through various mechanisms. Berberine acts as a natural PCSK9 inhibitor, which contributes to its hypolipidemic effects by enhancing the expression of LDLR and reducing PCSK9 expression and secretion, thereby promoting cholesterol clearance from the bloodstream [25, 29]. Clinical studies and meta-analyses have shown that berberine significantly reduces total cholesterol (TC), LDL, and triglycerides (TG) and increases high-density lipoprotein levels, either alone or in combination with other nutraceuticals [95]. The molecular mechanisms behind these effects include the downregulation of PCSK9 through pathways such as ERK1/2 signaling and the ubiquitin–proteasome degradation pathway. These mechanisms are also influenced by transcription factors like HNF1∝ and SREBP-2 [96, 97]. In animal models, berberine attenuates atherosclerotic lesions and hepatic steatosis by downregulating PCSK9 expression, further supporting its role in cardiovascular protection [96]. Moreover, the effectiveness of berberine on PCSK9 is enhanced when delivered using nanotechnology-based systems, which improves its pharmacokinetic profile and bioavailability [98]. However, some studies have reported that berberine can increase PCSK9 levels through SREBP-2 activation, suggesting a complex interaction that depends on the context and dosage [99].

### Tetrahydroberberrubine (THBru)

THBru, a derivative of berberine with higher bioavailability and lower toxicity, can improve hyperlipidemia by activating the AMPK/SREBP2/PCSK9/LDL receptor signaling pathway [100]. Feng et al. revealed that THBru effectively alleviates hyperlipidemia and obesity induced by a high-fat diet in mice by decreasing serum TC, TG, and LDL-C levels [100]. Molecular docking and cellular thermal shift assays mechanistically confirmed that THBru directly binds to AMPK with a binding energy of − 6.6 kcal/mol, activating this master metabolic regulator [100, 101]. Activation of AMPK by THBru leads to downregulation of SREBP2 and PCSK9 expression and upregulation of LDLR levels, thereby enhancing hepatic LDL clearance [16, 100]. In addition, THBru regulates cholesterol homeostasis by decreasing HMGCR, a rate-limiting enzyme in cholesterol synthesis, and increasing CYP7A1, which is key in cholesterol-to-bile-acid conversion [100, 102]. Notably, THBru reduces statin-induced PCSK9 expression, suggesting its potential as a combination therapy to enhance statin efficacy.

### Piceatannol (PT)

PT, a naturally occurring stilbenoid found in grapes, berries, and peanuts, shows potential in overcoming statin resistance in hypercholesterolemia through novel epigenetic mechanisms. PT effectively inhibits PCSK9 through p300 histone acetyltransferase (HAT) inhibition [30]. In vitro studies using HepG2 cells showed that PT suppresses statin-induced PCSK9 expression and stabilizes LDLR expression, leading to improved cholesterol clearance. Mechanistically, PT acts as a potent p300 HAT inhibitor, with an IC_50_ value of 19.89 μM. It competitively binds to the p300 HAT domain and blocks its recruitment to the PCSK9 promoter region, thereby preventing PCSK9 transcription [30, 103]. Unlike other PCSK9 inhibitors that act through transcription factors such as SREBP2 and HNF1α, PT regulates PCSK9 expression epigenetically, representing a unique mechanism of action. In vivo, high-fat diet-induced hypercholesterolemic mice coadministered simvastatin and PT for 10 weeks exhibited considerably reduced plasma LDL-C levels and enhanced hepatic LDLR stabilization compared with simvastatin monotherapy. These findings establish PT as a promising nutraceutical candidate for managing hypercholesterolemia, particularly in cases of statin resistance or intolerance, through its novel epigenetic control of the p300–PCSK9–LDLR axis [30, 48].

### Gallic acid (GA)

GA, a naturally occurring polyphenolic compound found in fruits, vegetables, and tea, can improve cholesterol metabolism by effectively regulating the PCSK9–LDLR axis. Zhang et al. reported that GA promotes LDL uptake in HepG2 cells through two mechanisms: enhancing LDLR protein accumulation via the EGFR-ERK1/2 signaling pathway and inhibiting PCSK9 expression [31]. In vitro studies showed that GA specifically binds to the extracellular domain of EGFR with a binding energy of − 6.97 kcal/mol, activating the EGFR-ERK1/2 pathway, which subsequently extends LDLR mRNA half-life from approximately 0.59 to 1.02 h, improving its stability and increasing LDLR protein accumulation [31, 104]. Mechanistically, GA also inhibits PCSK9 mRNA expression and protein accumulation through two distinct pathways: activating forkhead box O3 (FoxO3) and suppressing HNF1α, both of which are critical transcription factors regulating PCSK9 expression [31, 105]. Molecular docking studies further revealed that GA can directly bind to the active pocket of PCSK9 with a binding energy of − 5.36 kcal/mol, potentially blocking the PCSK9–LDLR interaction and reducing LDLR degradation. Therefore, GA can be envisaged as a promising natural compound for managing NAFLD associated with cholesterol metabolism disorders, particularly since cholesterol dysregulation is increasingly recognized as a key factor in NAFLD pathogenesis [106, 107]. Compared with pharmatherapy, GA offers advantages including minimal side effects, cost-effectiveness, and ready availability in common foods and beverages, particularly tea, where the GA content can range from 0.1 to 2% [108]. Owing to the multiple mechanisms through which GA improves LDL uptake, including the transcriptional regulation of PCSK9 and post-transcriptional stabilization of LDLR, GA is a valuable nutraceutical for cholesterol management and NAFLD prevention.

### Additional natural PCSK9 modulators

Several other natural compounds have demonstrated PCSK9-inhibitory properties through diverse mechanisms. Ellagic acid, a polyphenol abundant in fruits and nuts, markedly reduces PCSK9 expression and secretion while increasing LDLR protein levels through dual mechanisms involving HNF1α inhibition and FoxO3 activation, with dose-dependent effects confirmed in both HepG2 cells and ApoE−/− mice [105, 109]. Schisandrin A, from *Schisandra chinensis*, uniquely upregulates LDLR by destabilizing free PCSK9 protein in the proteasome rather than affecting transcription, thereby protecting LDLR from lysosomal degradation [110, 111].

Geranylgeranyl isoprenoids represent a novel mechanistic approach, as these mevalonate pathway-derived molecules regulate PCSK9 through the GGPP-Rap1a pathway. Wang et al. demonstrated that Rap1a activation enhances lysosomal degradation of PCSK9 and can suppress statin-induced PCSK9 elevation, suggesting potential for combination therapy [46, 112]. Plant extracts such as Welsh onion extract, containing kaempferol and p-coumaric acid, effectively attenuate PCSK9 and SREBP2 expression while maintaining LDLR levels [110, 113]. Similarly, pigeon pea (*Cajanus cajan* L.) extract reduces PCSK9 mRNA by up to 54% through HNF1α reduction, with cajaninstilbene acid identified as the primary bioactive component [114, 115]. Black raspberry extract demonstrates particular promise in combination with statins, counteracting statin-induced PCSK9 increases while providing additional cardiovascular benefits through gut microbiota modulation [116, 117].

Specialized plant-derived compounds include clerodane-type diterpenes from *Casearia grewiifolia*, *lindenane sesquiterpenoid* dimers from *Chloranthus japonicus* (particularly shizukaol F with 69.0% PCSK9 inhibition), neolignans from *Penthorum chinense* with IC50 values as low as 5.13 μM, and alkaloids from *Dendrobium nobile* that modulate multiple pathways including LXRα/IDOL/LDLR and SREBP2/HNF1α [118–121]. These compounds collectively demonstrate the rich diversity of natural PCSK9 modulators and their potential for developing novel therapeutic strategies for cholesterol management.

## Conclusions

The complex regulatory network controlling hepatocyte PCSK9 expression represents a promising target for the therapeutic treatment of hypercholesterolemia and associated cardiovascular diseases. This review highlights the diverse mechanisms involved inPCSK9 regulation, including transcriptional control by factors such as HNF1α/β and SREBPs, post-transcriptional modulation by microRNAs, epigenetic regulation through histone modifications, and modulation through multiple signaling pathways such as JAK/STAT, PI3K/Akt, and SOCS-3.

Natural compounds offer remarkable advantages as PCSK9 modulators, including greater accessibility and multitargeted actions that address various aspects of lipid metabolism. For instance, berberine and its derivative tetrahydroberberrubine acts on the PCSK9–LDLR axis through AMPK activation and ERK1/2 pathway modulation. Polyphenolic compounds like piceatannol offer novel epigenetic mechanisms by inhibiting p300 histone acetyltransferase, whereas GA and ellagic acid regulate PCSK9 through FoxO3 activation and HNF1α suppression. Plant extracts, such as those from Welsh onion, black raspberry, and *Dendrobium nobile* effectively counteract statin-induced PCSK9 elevation, suggesting their potential as complementary therapies.

The translation of these regulatory insights into nutraceutical applications represents a frontier in precise nutrition for cardiovascular health. Future research should focus on optimizing delivery systems to enhance bioavailability, determining effective dosages through rigorous clinical trials, and investigating potential synergistic effects between different natural compounds. In addition, personalized approaches that consider genetic variations in the PCSK9 pathway could maximize therapeutic outcomes.

By bridging fundamental molecular biology with translational nutritional science, natural PCSK9 modulators offer promising strategies to complement pharmatherapy, potentially increasing the accessibility of PCSK9-targeted therapies to broader populations and addressing the growing global burden of cardiovascular diseases. These nutraceutical strategies are particularly valuable for individuals with statin intolerance or those seeking integrative approaches to LDL-C management, though careful consideration of potential interactions and effects remains important given their comparable molecular mechanisms of action.

## Data Availability

No datasets were generated or analysed during the current study.

## Abbreviations

AMPK: AMP-activated Protein Kinase
ApoB: Apolipoprotein B
ApoE: Apolipoprotein E
ASCVD: Atherosclerotic Cardiovascular Disease
CAP1: Cyclase-associated Protein 1
CD36: Cluster of Differentiation 36
ChIP: Chromatin Immunoprecipitation
CK: Circular RNAs
CYP7A1: Cytochrome P450 Family 7 Subfamily A Member 1
DNLA: Dendrobium nobile Lindl. Alkaloids
EGFR: Epidermal Growth Factor Receptor
ERK1/2: Extracellular Signal-regulated Kinase 1/2
FASN: Fatty Acid Synthase
FoxO3: Forkhead Box O3
FXR: Farnesoid X Receptor
GA: Gallic Acid
GGPP: Geranylgeranyl Pyrophosphate
GSTP1: Glutathione S-transferase Pi 1
HAT: Histone Acetyltransferase
HDL: High-density Lipoprotein
HLA-C: Human Leukocyte Antigen C
HMGCR: 3-hydroxy-3-methylglutaryl-CoA Reductase
HNF1α/β: Hepatocyte Nuclear Factor 1 Alpha/Beta
IDOL: Inducible Degrader of LDLR
JAK: Janus Kinase
JNK: c-Jun N-terminal Kinase
LASER: Long non-coding RNA in Lipid Associated Single nucleotide polymorphism gEne Region
LDL-C: Low-density Lipoprotein Cholesterol
LDLR: Low-density Lipoprotein Receptor
lincRNA-p21: Long Intergenic Non-coding RNA p21
lncRNAs: Long Non-coding RNAs
Lp(a): Lipoprotein(a)
LRP-1: Low-density Lipoprotein Receptor-related Protein 1
LSD1: Lysine-specific Demethylase 1
LXRα: Liver X Receptor Alpha
MACE: Major Adverse Cardiovascular Events
MEK1: Mitogen-activated Protein Kinase Kinase 1
MHC-I: Major Histocompatibility Complex Class I
miRNAs: MicroRNAs
MLL1: Mixed Lineage Leukemia 1
NAFLD: Non-alcoholic Fatty Liver Disease
ncRNAs: Non-coding RNAs
NNT: Number Needed to Treat
NPAT: Nuclear Protein, Ataxia-telangiectasia Locus
OM: Oncostatin M
OSCs: Organosulfur Compounds
PCSK9: Proprotein Convertase Subtilisin/Kexin Type 9
PI3K: Phosphatidylinositol 3-kinase
PPARγ: Peroxisome Proliferator-activated Receptor Gamma
PT: Piceatannol
QALY: Quality-adjusted Life Years
Rap1a: Ras-related Protein Rap-1A
ROCK: Rho-associated Protein Kinase
SET1/COMPASS: Set1/Complex of Proteins Associated with Set1
siRNA: Small Interfering RNA
SIRT1: Sirtuin 1
SOCS-3: Suppressor of Cytokine Signaling-3
SRE: Sterol Regulatory Element
SREBP-1c/2: Sterol Regulatory Element-binding Protein-1c/2
STAT3: Signal Transducer and Activator of Transcription 3
TC: Total Cholesterol
TG: Triglycerides
THBru: Tetrahydroberberrubine
TRRAP: Transformation/transcription domain-associated Protein
